# Supplementary material for: The causal role of circulating inflammatory markers in osteoporosis: a bidirectional Mendelian randomized study
Source: Front Immunol. 2024 Jul 18;15:1412298. doi: 10.3389/fimmu.2024.1412298 (PMC11291241; doi:10.3389/fimmu.2024.1412298)
Supplement: Supplementary file 1 [file DataSheet_1.zip › supplementary material/OSTEOPOROSIS-CIM/GCST90274772.tsv.gz_forest.pdf]

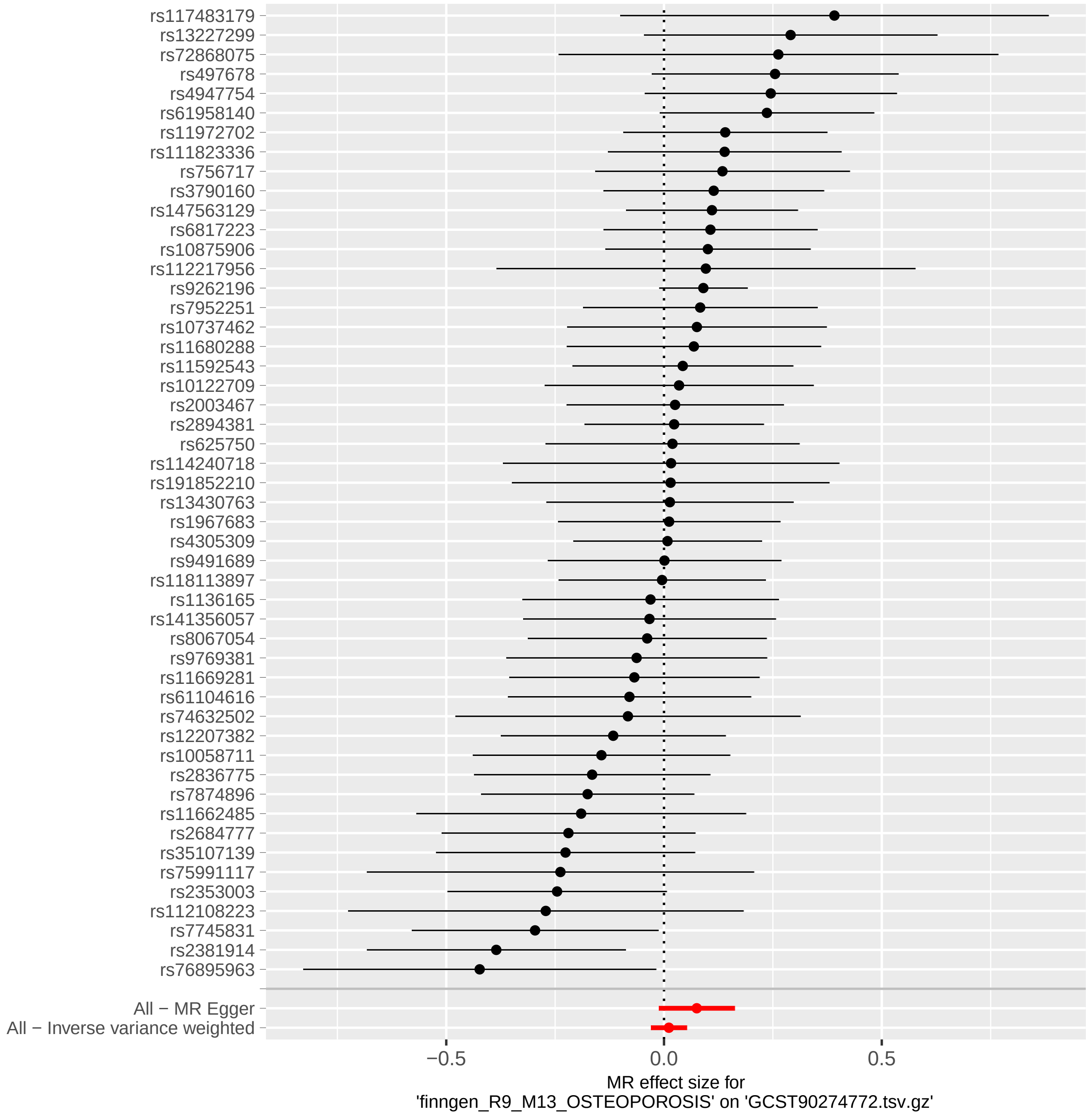

rs117483179
rs13227299
rs72868075
rs497678
rs4947754
rs61958140
rs11972702
rs111823336
rs756717
rs3790160
rs147563129
rs6817223
rs10875906
rs112217956
rs9262196
rs7952251
rs10737462
rs11680288
rs11592543
rs10122709
rs2003467
rs2894381
rs625750
rs114240718
rs191852210
rs13430763
rs1967683
rs4305309
rs9491689
rs118113897
rs1136165
rs141356057
rs8067054
rs9769381
rs11669281
rs61104616
rs74632502
rs12207382
rs10058711
rs2836775
rs7874896
rs11662485
rs2684777
rs35107139
rs75991117
rs2353003
rs112108223
rs7745831
rs2381914
rs76895963
All − MR Egger
All − Inverse variance weighted
−0.5
0.0
0.5
MR effect size for
'finngen_R9_M13_OSTEOPOROSIS' on 'GCST90274772.tsv.gz'
